# A qualitative interview study exploring the experiences of pain specialists on prescribing opioids for chronic non-cancer pain

**DOI:** 10.1038/s41598-025-15113-6

**Published:** 2025-08-13

**Authors:** Thomas F. Kallman, Emmanuel Bäckryd, Anne Söderlund Schaller

**Affiliations:** Pain and Rehabilitation Center, and Department of Health, Medicine and Caring Sciences, Linköping University, Brigadgatan 22, Linköping, SE-581 85 Sweden

**Keywords:** Chronic pain, Opioid prescribing, Qualitative methodology, Interview study, Sweden, Pain physician, Pain management, Chronic pain, Health services

## Abstract

Opioid prescribing for patients with chronic non-cancer pain is common despite issues associated with long-term efficacy, functional improvement, and safety. Pain specialists assess many patients with chronic non-cancer pain, but their experiences of this situation are not well represented in prior qualitative research. The aim of this study was to explore pain specialists’ experiences of prescribing opioids to patients with chronic non-cancer pain. We adhered to the Consolidated Criteria for Reporting Qualitative Research guidelines. Pain specialists in Sweden were recruited by purposive and snowball sampling. Participants were digitally interviewed, audio and video were recorded, and interviews translated verbatim. Data was analyzed through manifest inductive content analysis. Twenty pain specialists were interviewed. Qualitative content analysis revealed that the pain specialists’ experiences were represented by two main categories: (1) Navigating the doctor-patient relationship, and (2) Challenges and opportunities when prescribing opioids. The first main category describes the relational demands associated with opioid prescribing and includes communication, conflicts, managing expectations, and the emotional and ethical aspects of prescribing opioids. The second main category describes handling complexity and heterogeneity, organizational aspects, and the doctor’s due diligence when prescribing opioids. Our results offer new insights into pain specialists’ experiences of prescribing opioids for chronic non-cancer pain, offering health care professionals guidance for responsible opioid prescribing. Pain specialists highlight the need for structured pain assessments and identify system-level improvements, such as allocating sufficient time, enabling team-based care, and continued education initiatives, to support safe opioid use in health care.

**Supplementary Information:**

The online version contains supplementary material available at 10.1038/s41598-025-15113-6.

## Introduction

The global prevalence of chronic pain ranges between 20 and 30%^1,2^. It is a burdensome condition, affecting many aspects of life^3^ and often leading to low physical functioning, low quality of life, and high psychological distress^4,5^. When treating moderate-to-severe acute pain, e.g., severe traumatic injuries or in an inpatient perioperative setting, opioids are commonly used for a short period and are usually effective^6,7^. For chronic non-cancer pain (CNCP), however, the situation is different. Opioids prescribed for CNCP seldom lead to substantial pain relief^8^ and they do not (on group-level) yield long-term, clinically meaningful improvements in quality of life^9^. Consequently, treatment recommendations emphasize optimizing non-pharmacological treatments and non-opioid analgesics, keeping opioids as a last resort for select patients with CNCP after performing a risk-benefit analysis^6,10,11^. Despite these recommendations, about one-third of CNCP patients referred to specialized pain care in Sweden use opioids^12,13^. This is concerning from guideline-adherence and patient safety perspectives, particularly when considering the serious adverse events associated with long-term use of opioids, such as overdose or addiction^14,15^.

A recent Swedish study found that 66% of patients with long-term opioid treatment assessed in specialized pain care were referred from primary health care^16^. Hence, general practitioners (GPs) assess many patients with CNCP and concomitant opioid treatment. Many previous qualitative studies have explored GPs’ experiences when prescribing opioids for CNCP^17–19^ including emerging research regarding physicians’ experiences when deprescribing^20–22^. A systematic review of qualitative evidence summarized GPs’ experiences into four themes: (1) caught in the middle of the opioid crisis; (2) ‘are opioids always bad?’; (3) the GPs’ weighing scale, accounting for patient-related and therapeutic relationship-related factors; and (4) sense of powerlessness due to lack of alternatives, time, and support from specialists^23^.

Given how many patients with opioid treatment pain specialists regularly assess^16^ there is a general lack of representation of pain specialists’ experiences of opioid prescribing for CNCP in current literature. One previous qualitative study explored what makes opioid prescriptions become long-term, but only a minor proportion of participants were pain specialists^24^. Another study, from Thailand, examined pain specialists’ perspectives but also included patients and their family members and did not specify criteria for the pain specialists^25^. Pain specialists expressed heterogenous attitudes about prescribing opioids: using it as a last resort in select patients, avoidance, frustration, comfortable prescribing long-term, reasoning about potential benefits, effectiveness, and concerns associated with opioid treatment such as adverse effects and opioid use disorder, as well as highlighting the importance of prescribing policies^25^.

While the paper by Seangrung et al.^25^ offers needed insight into pain specialists’ perspectives on prescribing opioids for CNCP, there remains an aforementioned lack of studies on the topic in general, and in a Swedish context in particular. Thus, the aim of the current study was to explore Swedish pain specialists’ experiences of prescribing opioids to patients with CNCP.

## Methods

The Consolidated Criteria for Reporting Qualitative Research (COREQ) guidelines were used to guide the design and reporting of this inductive qualitative interview study^26^. To facilitate exploration of the participants own experiences of prescribing opioids for CNCP, we used open-ended questions and qualitative content analysis methodology as described by Elo and Kyngas^27^ and Krippendorff^28^.

### Participants and recruitment

The formal requirements for board certification in pain medicine in Sweden are regulated by the National Board of Health and Welfare (NBHW) in regulations SOSFS 2015:8 and HSLF-FS 2021:8. Physicians who already are specialists in a so-called clinical base specialty with extensive direct contact with patients (i.e., excluding for instance laboratory medicine), can sub-specialize in pain medicine. Completed sub-specialization in pain medicine (i.e., board certified as “pain specialist”) was therefore used as the inclusion criteria.

Twenty-one geographical regions in Sweden are responsible for the health care in each region. In turn, these 21 regions are grouped into six overarching health care regions from which participants were recruited^29^. Sweden has a population of 10 million, and at study start there were about 150 pain specialists working clinically in Sweden^30,31^. Hence, Swedish pain specialists meet a selected population of pain patients. We considered that author TFK was known by pain specialists at the Pain and Rehabilitation Center (PRC) in Linköping and that this knowledge could impact the interview situation. Therefore, pain specialists employed at the PRC in Linköping were excluded from participation. Thus, we estimated that about 140 pain specialists were available for recruitment and set out to interview 20 pain specialists.

Based on our research group’s knowledge of Swedish pain specialists, we used purposive sampling and contacted pain specialists directly by email or through face-to-face recruitment at the Swedish Pain Forum in Linköping 2022. After interviews were completed, interviewees were asked if they knew of other pain specialists who they believed could be interested in participating in the current study. Thus, we also used snowball sampling for recruitment of participants.

###  Interview procedure and transcription

A first draft of the interview guide was developed based on the research group’s clinical experience and research knowledge on the topic being studied. This interview guide was tested in a pilot interview in June 2022. After transcription, authors TFK and AnS read the transcript separately and then conferred together. Small adjustments were made to a few questions in the interview guide to better reflect the aim of the study. The pilot interview was included in the final data analysis, as it provided data of sufficient quality and relevance to the study aim. Interviews were semi-structured with open-ended questions to facilitate answers that reflected the participant’s own experiences. The interview guide was used in all interviews, and field notes were noted on the interview guides during the interviews, to ensure that all questions and aspects of the study topic were included in each interview. After gathering initial demographic data, each interview began with the same question: “How do you experience prescribing opioids to patients with chronic non-cancer pain?”. A translated version of the interview guide is available in the Supplementary Information.

All interviews were conducted digitally via Zoom, in Swedish, by author TFK between June 2022 and February 2024. Audio and video were recorded. Each interview was done once and began in the same manner: the interviewer introduced himself with name, occupation, place of employment, and reiterated the study aim and interview topic. Only the interviewer and participant were present during the interview. Written study information had been sent to all interviewees prior to the interview. After the interviews were completed, the program software Zoom automatically saved audio and video files to the computer used during the interviews. Interviews were therefore stored digitally and TFK had access to the audio and video files. To enable the six first interviews to be transcribed by medical secretaries at the PRC in Linköping, the corresponding audio files were uploaded to a specific folder on Region Östergötland’s secure internal server system, thereby ensuring participant confidentiality.

All interviews were transcribed verbatim in Swedish. The six first interviews were transcribed by medical secretaries at the PRC in Linköping. Due to organizational changes at the PRC, the 14 remaining interviews were transcribed by Transkriptor^32^ which is an artificial intelligence-based, online transcription software which is HIPAA compliant, ensuring participant confidentiality. Author TFK then listened through all interviews, in parallel read the transcripts, and edited any errors found in the transcripts. If it was not possible to ascertain what was said at a specific point in an interview (e.g., due to temporary poor internet connection), the word or sentence was omitted and the reason for the omission was specified in the transcript.

### Data analysis

This study is based on an epistemological perspective where healthcare professionals’ experiences of opioid prescription is understood as a context-dependent knowledge that is shaped by the participants’ own narratives. A manifest content analysis was used to describe the explicit content of these narratives, focusing on what was said rather than underlying meanings. Latent content was therefore not included in the analysis. Ontologically, we assume that reality is constructed through individuals’ experiences and interpretations, implying that there is no single objective truth to uncover regarding opioid prescribing, but rather multiple possible understandings^33^.

To analyze the data, we employed inductive qualitative content analysis as outlined by Elo and Kyngäs^27^ which facilitates a systematic identification and abstraction of meaning units from interview transcripts. More specifically, this process entailed repetitively and systematically reading the interviews to find recurring words, sentences and themes which were relevant for the study aim. Authors TFK and AnS read each interview independently from one another. Author TFK was responsible for open coding, sub-categorization of codes, and abstraction of the sub-categories to categories and main categories; this was done in NVivo (version 15) and Microsoft Word (version 16). After completion of each step in the above-mentioned analysis process, results were discussed with AnS, consensus was reached, and TFK revised the results as needed. Results were then sent to author EB who independently analyzed the results and provided feedback to TFK and AnS. When consensus regarding the analysis was reached, the results were translated from Swedish to English by TFK and thereafter revised by EB and AnS. If a quote was taken from the middle of an ongoing sentence or paragraph, this was denoted in the quotes as […]. In the case a quote needed context for the sake of readability or clarity, we added these words in brackets.

We chose an inductive approach due to (1) a general lack of prior qualitative studies conducted on pain specialists’ experiences of opioid prescribing; (2) an assessment that this approach would more accurately reflect the pain specialists’ experiences; and (3) we assumed that the context in which the pain specialists’ opioid prescribing takes place was different compared to the GPs, which we viewed could influence the experiences which pain specialists have experienced, thus further motivating an inductive approach. The goal was to capture patterns in participants’ experiences rather than to produce generalizable results. The researcher assumes an active interpretive role while striving to ensure transparency and trustworthiness in representing the participants’ voices.

### Research team and reflexivity

During the study period, author TFK who conducted all interviews worked as an intern and later as a resident physician and had some clinical experience of assessing patients with CNCP and opioid treatment, as well as basic knowledge of qualitative methodology. Author EB is a pain specialist with extensive clinical experience of opioid-treated patients. EB is also a senior associate professor in pain medicine with prior opioid-related research and basic knowledge of qualitative methodology. Author AnS is a specialized pain nurse with extensive experience caring for patients with chronic pain and has extensive knowledge regarding qualitative methodology with prior publications using the same methodology used in the current study^34,35^.

### Ethics

Ethical approval was granted by the Swedish Ethical Review Authority (Dnr 2022-05169-01). All methods were performed in accordance with relevant guidelines and regulations. Written information about the study, data management, and the participants’ rights as research participants were sent to all participants prior to the interview. The written information stated that participation was based on the participants’ own free volition and that they at any point in the research process could discontinue their participation, if they desired. All participants were asked to give written informed consent prior to the interviews. To ensure that all participants were interviewed voluntarily, each interview began with a confirmation that the participant had provided written informed consent via the digital form. If a participant had not filled out the digital consent form, this was addressed at the beginning of the video call. The participant was again asked if they had read the study information and if they consented to participation, and if verbal consent was given the interview proceeded.

### Patient and public involvement

Neither patients nor the public were involved in the design, conductance, or reporting of the present study. Neither transcripts nor results were sent to participants for checking.

## Results

Twenty-six pain specialists were asked to participate and twenty-two (85%) agreed to be interviewed. Of the four who did not participate, two did not respond to participation inquiries, one had not finished specialization, and one declined to participate. Two pain specialists who agreed to participate did not set a date for the interview and were subsequently excluded. Thus, 20 pain specialists (77%) were interviewed. During one interview, the participant informed the interviewer that they lacked board certification but had over 20 years of experience in specialized pain care. After discussing this in the research group and reading the interview transcript, we considered this participant to be the equivalent of a pain specialist.

**Fig. 1:**
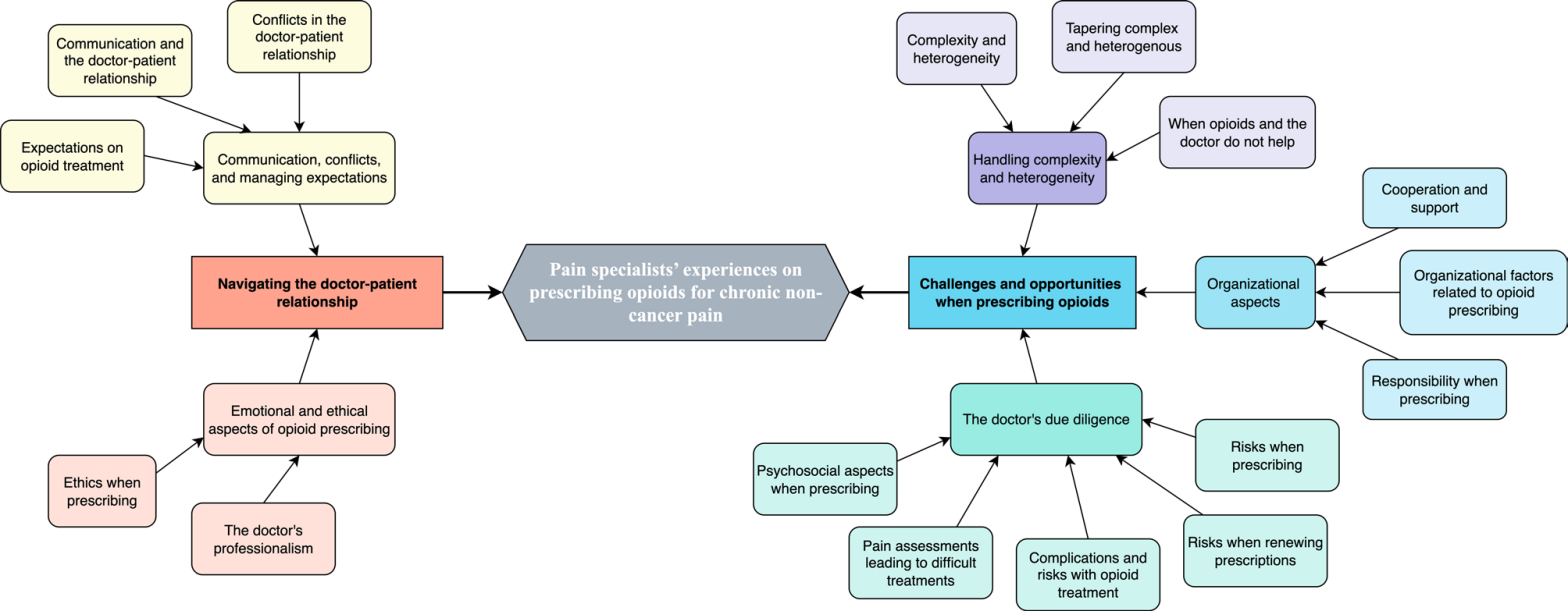
The abstraction process from sub-categories, to categories, and finally main categories in relation to the study aim.

Descriptive data of the 20 pain specialists who participated are presented in Table 1. Figure 1 shows the abstraction process from sub-categories, to categories, and finally main categories in relation to the study aim. The two main categories are expounded below.

**Table 1:** Descriptive data of participants. All values presented are either median (min-max) or percentage.

Variable	Result
Interview duration (minutes)	43 (31–66)
Age (years)	57 (45–72)
Sex (% male)	55
Years experience as physician	30 (17–47)
Years experience as pain specialist	11 (0–28)
Base specialty (%)	
General practitioner	20
Anesthesiology	45
Internal medicine	5
Orthopedic surgery	5
Neurology	5
More than one specialty	20
Overarching health care region (%)	
Mid Sweden	40
Stockholm-Gotland	20
Western	10
Southeastern	20
Southern	10

### Navigating the doctor-patient relationship

This category describes the participants’ experiences of managing the relational demands which opioid prescribing places on them whilst managing their own individual proclivities.

#### Communication, conflicts, and managing expectations

Most participants viewed the doctor-patient relationship and communication as central aspects when prescribing opioids. Factors described to facilitate the doctor-patient relationship were continuity, opioid contracts, and compromising in certain situations, e.g. in elderly patients, while simultaneously being observant of warning signs exhibited by patients. Indication, effects, differing opinions, and clarifying the framework for opioid treatment were seen as important topics to discuss, which were perceived to build mutual trust and a treatment alliance. This could give some participants the mandate to make changes in the patients’ treatment, while others described that medically motivated changes caused some patients to be displeased. Patients’ attitudes and thoughts regarding their treatment were actively explored by participants, who often experienced a discrepancy between the patient’s expectations of pain relief and the specialists’ knowledge of pain medicine’s limitations. Nearly half of the participants saw opioid prescribing as being an integral part of their professional role but noted that in some situations, physicians enter into an already established doctor-patient relationship, for example when assuming responsibility for a colleague’s patient.

*“[…] you sort of enter a… a role*,* an established role. Kind of like the doctor who had many patients who only came to get morphine prescribed. Those are the roles that were established. The younger [doctors] question it. They wonder*,* how come*,* why does this [patient] come here and receive morphine? Yes*,* but don’t you care about that*,* now you’re the one prescribing. And worst-case scenario you accept that and*,* in the end*,* accept responsibility for these [patients].”* Participant 10.

Participants noted that patients’ behaviors and attitudes, such as help-seeking or fear of opioid discontinuation, were patient-related factors which could influence the prescribing situation. Some participants described that patients felt opioids were the only thing that helped them and that they therefore had a right to the medication, and that this was a situation which could be difficult to manage. One participant opined that patients knew opioids did not help them. More than half of the participants described receiving threats or experiencing conflicts when prescribing opioids, with some patients demanding opioids. One participant noted that such demands rarely were put forward for other types of medications. Almost half described a changed balance of power in the doctor-patient relationship, expressed as feeling powerless or as though the patient was in control of the treatment. Some likened it to an extortion situation with patients expressing different threats if the pain specialist chose to not prescribe: reporting participants to review boards, indirect suicide threats, and blaming the participants for the consequences the patient would experience if the demand to prescribe was not met. Some participants described heterogenous outcomes when attempting to resolve these conflicts, and one participant considered it was not feasible to engage in every conflict. Documentation in medical records was viewed as essential for effective communication with patients and for protecting pain specialists in the event of a regulatory review.

*“And I*,* unfortunately*,* say that it’s about the same as if a cancer patient would say to their oncologist – I have the right to be rid of your*,* of my cancer. The patient has the right to receive the best possible treatment. The patient has the right that the doctor really*,* really tries to the best of their ability. But to succeed*,* that medical science or the art of medicine has the possibility to then actually do it*,* that is a completely different story. Unfortunately*,* patients still die from cancer. Unfortunately*,* patients still die from heart attacks and the like. And unfortunately*,* incurable pain exists.”* Participant 20.

#### Emotional and ethical aspects of opioid prescribing

Most participants mentioned that personal characteristics such as medical competence, preconceived notions, ability to set boundaries and manage personal resources could affect opioid prescribing. Most described having been emotionally affected by prescribing, mentioning frustration over patients disregarding plans; anger when other doctors prescribed opioids to patients who previously had been denied a prescription; discomfort and fear in the prescribing situation; concern for the patients’ future; guilt and shame that patients had started opioid treatment; regret that they started prescribing; feeling forced to prescribe; and the difficulty associated with making patients feel dissatisfied.

*“Powerlessness. Anger. Irritation. And sorrow. Over that I knew that we had something to offer that*,* that would be*,* would be able to be*,* yes*,* a long-term solution for this patient also. But there was such an exaggerated belief that medications would help her.”* Participant 19.

To become comfortable with handling opioid-related questions and avoid becoming emotionally invested in the prescribing situations, more than half of the participants considered that physicians needed to assess many patients and described that it became easier over time. Participants also noted that their attitude towards opioids shifted as their experience increased: from initially having been taught that life-long treatment with opioids could be a viable option, to becoming more restrictive. One-third considered that the habit of prescribing opioids could be considered a form of avoidance behavior, and some participants mentioned feeling ambivalent when prescribing in certain situations.

*“[…] for example*,* patients with endometriosis frequenting the emergency department and receiving oxycodone-injections. And seek medical care*,* like*,* multiple times per month. I start those patients on buprenorphine patches or tapentadol instead. And if the behavior ‘seek emergency medical care’ drastically decreases*,* I believe I’ve justified a long-acting opioid. But I think it’s wrong. […] Because I usually think that I’m treating a nociplastic pain with*,* with opioids.”* Participant 16.

Almost all participants had encountered ethical questions related to the understanding of human nature, autonomy, or duty ethics when prescribing opioids. Half of the participants mentioned the do-no-harm principle as central. Opioids were considered to potentially contribute to a helping ethic, but that the nature of opioids made them particularly challenging for the prescriber. Participants described sometimes having a tough approach and conditioning prescriptions. Simultaneously, in some situations the participants considered they did the least amount of harm and prevented worse consequences by continued prescribing. About half of the participants had experienced that the suffering expressed by patients could influence their choices when prescribing.

*“You have an ethical assessment where you think this is wrong. Yet you still agree to it because*,* well*,* right now might not be the right time for the patient because they’re feeling sad*,* it’s been difficult*,* and it hasn’t succeeded previously. Alright*,* we’ll prescribe*,* and they can continue. And then you’ve contradicted your own ethics that you previously presented so nicely. And then you go home with*,* with a sour feeling instead… […] that’s why you don’t want to see these types of patients again*,* because they cause so much suffering for yourself. It’s so hard to sit and act against your own better judgment*,* or*,* or own opinion.”* Participant 10.

### Challenges and opportunities when prescribing opioids

This category describes both the challenges the participants have faced when prescribing opioids and the areas in which they see opportunities for improvement.

#### Handling complexity and heterogeneity

Most participants described the prescribing situation as heterogenous: sometimes easy, sometimes difficult. Challenges included the subjective nature of pain, that some patients were perceived to be ‘stuck’ in their treatment and unwilling to change their treatment while others wanted to stop, difficulties knowing which patient could benefit from opioids, and that physicians could assess the same situation differently, leading to different prescribing decisions. One participant noted that the issues brought up by patients frequently followed stereotypical patterns. One-fourth of participants saw a need to individualize opioid treatment based on what works for the patient and their attitude towards opioids, but that it was a balancing act as participants considered opioids a double-edged treatment. Most described that opioids could hinder rather than help patients and continued opioid treatment was considered to hinder pain rehabilitation efforts. Participants expressed awareness of less common adverse effects, such as opioid-induced hyperalgesia and endocrinopathies. One-third of participants noted that physicians’ desire to help can lead to a persistent, and at times excessive, effort to find solutions for the patient. This was, in turn, considered a potential risk for losing control over the prescribing situation.

*“[…] I think that it’s with good intentions that you believe you’re helping the patient. And that we have*,* I think most of us in health care have a large empathetic way of thinking*,* that we want to try to help the patient.”* Participant 19.

All participants described experiences of tapering opioids. This was seen as a heterogenous situation for both patient and prescriber. For the former, heterogenous attitudes and outcomes were underlined. For prescribers, tapering was perceived to be a stressful and challenging situation, particularly in patients with long-term treatment who were described as difficult to motivate. Tapering was viewed as leading to an initial increase of pain intensity. However, if tapering continued, participants described often seeing improvements in the patients’ overall disposition, energy levels, or cognitive function, and that patients could resume daily life activites and sometimes even return to work. A few participants described the effects of opioid tapering on overall patient status as potentially surpassing those seen in pain rehabilitation programs. Not all opioids were considered as easy to taper as others. Participants emphasized the importance of doing one thing at a time, that the patient’s own motivation was paramount, and that tapering should be tailored to the individual. A minority of participants described meeting patients who had gone through forced tapering, whether done by other physicians, colleagues, or the participants themselves. Forced tapering was viewed as a poor approach in part because it could be a traumatic experience for the patient. Participants emphasized the importance of respecting the fact that tapering-induced withdrawal is a challenging experience for patients.

*“Most patients that I see*,* they already have opioid-related problems – that’s when I meet them. And that’s a group that can behave in very different ways. They are different*,* these patients […] Then there’s a group of these patients who are ambivalent – on the one hand*,* on the other – you know. They get that it’s not good on the other side*,* they need to work*,* they need to be able to care for their children*,* maybe*,* or move around […] And then there are those who*,* of course*,* are completely anti and who think I’m crazy if I say they would need to taper their opioids.”* Participant 6.

#### Organizational aspects

Opioid prescribing guidelines were positively viewed by most participants. They thought guidelines could influence the prescriber’s behavior and help them handle problems which may arise when prescribing opioids. Simultaneously, participants pointed out problems such as that guidelines often were not adhered to and did not encompass all patients. A well-organized health care system, adequate pain and primary care resources, well-functioning IT-systems, and having competent people in leadership roles were seen as important factors which could facilitate or hinder responsible opioid prescribing. Many participants described a lack of knowledge regarding various aspects of opioid prescribing amongst physicians, health care staff, patients, and in society. This was considered to contribute to the challenges which many participants had experienced when trying to educate patients, and they saw a need for opioid education efforts on multiple levels. Almost half of the participants mentioned the importance of making responsible opioid prescribing a medical priority, and a few had experienced that this topic was not prioritized by executive leadership. Participants viewed leadership as a central aspect for influencing opioid prescribing practices and for implementing guidelines, noting that a lack of medical competence in leadership could result in inaccurate prescribing guidelines.

*“[…] I’ve educated primary care centers and then there was a private one where economists decided that buprenorphine patches were too expensive and instead you could prescribe fentanyl. Seriously. And*,* eh…then I argued*,* what are you doing? ‘Yes*,* but we prescribe fentanyl instead’. But hello*,* medically it’s totally wrong. You can’t do that.”* Participant 6.

Most participants described challenges regarding formal responsibility when prescribing opioids, for example if multiple health care units were involved. Some described a general attitude within health care not to take over opioid prescribing which had been started by other health care clinics, while others emphasized it was important to try to help. Difficulties could also arise when transferring the formal responsibility between physicians working at the same unit, and the participants viewed that this was a common challenge within primary care. Two-thirds of participants saw a need for improved collaboration with primary care and other specialist clinics, such as surgical, psychiatric, and addiction medicine clinics. Almost half of the participants mentioned the referrals that their pain clinics received, referrals which often were about opioid-related problems or failed tapering. Most participants emphasized that sufficient time was foundational for responsible opioid prescribing, often requiring frequent follow-ups which could be time consuming. The possibility of working in a team, for example with nurses who could assist with the frequent follow-ups, as well as being able to discuss, tutor, and support physicians in similar situations were described as important when prescribing opioids.

*“[…] we’ve had to be*,* perhaps somewhat extreme for the signal value*,* right? A little bit. But*,* in accordance with that*,* is the fact that I would never dream of referring any of my patients to a general practitioner and ask them to take over. Why would they say yes to that? I would never say yes to take over if they messed up. It must go both ways.”* Participant 8.

#### The doctor’s due diligence

More than half of participants considered that a thorough pain assessment was foundational for prescribing opioids. Participants described that the treatment indication often could be questioned, was unclear or absent, and that the pain assessment could be used as a tool for discussing the ongoing treatment. Participants emphasized the need to think mechanistically and to not assume that all chronic non-cancer pain was nociplastic in mechanism. One-third of participants noted the biopsychosocial context in which opioid prescribing occurs. Patients could be subject to vulnerable social situations or have psychiatric co-morbidities, which participants viewed could impact their own room for action. Half the participants described that patients didn’t need opioids for their pain condition, but rather that the treatment continued due to some of the other effects of opioids. Some participants saw that patients had low quality of life and a non-functional daily life. Patients who were on high opioid doses, but still functioned well in everyday life, were perceived as particularly challenging to assess.

*“[…] on a five-point scale where*,* where five corresponds to hard training and one is bedridden*,* you’re at one to two*,* that’s fairly common. So*,* I wonder*,* where did your life go? Was this how you imagined your life? That you would have this level of activity? No*,* of course you didn’t. No*,* but… you thought that the medication would help you*,* but I see that the medication hasn’t helped you even a single day to any better activity level. ‘No*,* but if I didn’t have it then I wouldn’t even get out of bed.’ No*,* but… it’s still not leading you in the direction you desire.”* Participant 10.

Almost all participants mentioned the importance of conducting a risk-benefit analysis (short- and long-term), taking into consideration factors such as the risk for dose escalation, overdose, and addiction. Most participants expressed worry that their patients would become addicted and described some patients as being “kidnapped” by opioids. Participants had met patients with mixed substance abuse and noted that some patients turned to illicit use, for example by purchasing opioids through the internet. The addiction potential associated with opioids caused many participants to question if these complex situations could be handled within primary care. One-fifth of participants described the stigmatization that patients with chronic pain and opioid treatment could be subject to and that these patients could slip through the cracks in health care. More than half of participants regarded the prescription renewal situation as challenging and time-consuming, with one participant describing that renewing a prescription goes very quickly, whereas saying no requires much more effort. This situation often made participants feel suspicious, especially if the patient had certain co-morbidities, if the pain specialist had “inherited” the patient from a colleague, if the patient had deviated from the treatment plan, or if the patient used specific opioids such as fentanyl. Many participants pondered the fact that opioids are classified as narcotic drugs and described that some patients had tricked them and sold their opioids, lost prescriptions, or had increased doses on their own, leading to premature prescription renewal requests.

*“[…] it’s important to have very good control over it. And*,* like*,* you can’t get a picture of it when you’re going to renew a prescription on a Friday afternoon. You have no idea how things are for this patient*,* really*,* and if it’s*,* is this reasonable or not? The only thing you see is that someone is desperate and that they’ve used their pills or was it patches or whatever it was*,* way too quickly.”* Participant 9.

## Discussion

In this study we found that Swedish pain specialists’ experiences and perspectives on prescribing opioids for CNCP were encompassed by two main categories, discussed below.

### Demands of the opioid-laced doctor-patient relationship

The experience of pain is subjective and what constitutes ‘acceptable pain’ may vary between individuals with chronic pain^36,37^. Within this context, in most scenarios, the prescription of opioids is the physician’s well-intended attempt to treat the patient’s pain. The conflicts which participants experienced when communicating with patients about opioids are therefore understandable. Hence, the emphasis placed on building mutual trust in the doctor-patient relationship through communication and managing expectations as evidenced by our results in subcategory 3.1.1 is understandable, considering communication can improve patient-centered care for patients with chronic pain^38^. Unfortunately, receiving threats or feeling extorted by the opioid-treated patient with CNCP when prescribing opioids seems to be an aspect in subcategory 3.1.1 which the prescriber must learn to handle. Given these experiences, it is unsurprising that opioid prescribing for CNCP may introduce a change in the balance of power in the doctor-patient relationship. In a manner similar to what is the case for GPs^23^ our results indicated feelings of powerlessness in the opioid prescribing situation, sometimes due to clashing expectations between patient and prescriber, and that managing this was demanding.

Many patients suffering from CNCP have low levels of physical functioning and quality of life^4,5^ and opioid treatment does not seem to improve these factors^9,12^. Hence, it is understandable that the ethical and emotional aspects identified in category 3.1.2 may arise when pain specialists are tasked with assessing if the opioid-treated patient with CNCP should continue their treatment or not. Given that the do-no-harm principle was a central aspect when assessing opioid treatments, it is understandable that pain specialists, similarly to GPs^18^ may experience distress and perhaps even develop an avoidance-based attitude if they feel pressurized to act against their professional ethical code, medical knowledge, and personal convictions. The ability to feel empathy for people in pain is vital, but may come at the cost of poorer mental health^39^ and such aspects may be present in the opioid prescribing situation for CNCP. GPs have expressed feeling ill-equipped to handle opioid prescribing and therefore wanting to avoid opioid prescribing altogether^40^ rather than wanting to avoid it due to ethical dilemmas. Regardless of if the patient is assessed in primary or specialized pain care, repeated exposure leading to increased clinical experience seems to be a fundamental key for developing the skills to manage the demands which opioids introduce into the doctor-patient relationship. However, our results indicate that opioid-treated patients suffering from CNCP may be better cared for in specialized pain care compared to primary care, particularly if serious opioid-related problems have begun to emerge. If it is not possible for pain specialists to take over CNCP opioid prescribing altogether, then at least the structure of primary care should be improved and GPs given clear pathways to consult pain specialists regarding these patients.

### Cornerstones of safe opioid prescribing

Category 3.2.3 borrowed its name from the business world, as due diligence for companies seeking to merge and acquire other companies often is an important yet strenuous and time-consuming process^41^. Similarly, structured clinical reasoning summarized in a pain assessment^42^ which accounts for the patient’s biopsychosocial context and includes a risk-benefit analysis of the opioid treatment, can be a strenuous and time-consuming activity when dealing with opioid-treated CNCP patients. Nevertheless, our results emphasized pain assessments as essential for managing the complexity of prescribing opioids. The risks associated with long-term opioid treatment^14^ such as dose escalation and overdose, combined with the facts that as many as one-fifth of opioid-treated patients may exhibit aberrant behavior and about 10% suffer from opioid use disorder^43^ further strengthens the argument for structured, biopsychosocial pain assessments^42^. The pain mechanism of chronic primary pain conditions such as, e.g., fibromyalgia, is nociplastic, and opioids are not recommended for such conditions^10,44^. Yet roughly one-third of patients referred to specialized pain care in Sweden use opioids^12,13^. There seems to be an educational need in primary care concerning how to assess chronic pain. Such educational endeavors could influence prescribing habits and lead to improved identification of opioid-related problems and, ultimately, to safer opioid prescribing.

A crucial aspect of opioid prescribing is the ability to determine when tapering is appropriate^45^. Our results also indicated that when the prescriber has chosen this option, then it is important to remain steadfast in this decision and help the patient cope with the initial withdrawal-associated increase in pain intensity^46^. There is evidence, albeit currently of low quality, that opioid tapering improves pain^47^ function, and quality of life^48^ and our results are in line with this. Due to the risks of forced tapering^49,50^ the participants’ emphasis on tailor-made tapering in a well-motivated patient seems appropriate^51^. Our results further indicated that pain specialists were aware of less well-known adverse effects such as opioid-induced hyperalgesia and endocrinopathies (e.g., hypogonadism) – conditions for which tapering can be a cure^52^.

### System level opportunities to facilitate opioid stewardship

Multiple prescriber-situations are inherently problematic in this setting^53^ and our results confirmed that these situations could be challenging to handle. This system level challenge may arise when multiple clinics and/or multiple prescribers are involved. To mitigate this, structural health care system reforms are required. Having medically competent individuals in leadership may be an important key to effectuate meaningful change in this regard. Conversely, our results identified that lack of medical competence in leadership seems to be a risk factor for implementation of medically incorrect prescribing practices. This is particularly problematic, as it is known that guidelines may affect prescribing habits^54^ yet implementation of opioid stewardship falters^55^. According to nationwide publicly available aggregated statistics from the NBHW in Sweden, the yearly prevalence of dispensed opioids in Sweden has fallen by about 20% during 2014–2023, albeit with a dramatic shift in choice of opioid^56^. Our results in subcategory 3.2.2 recognized that opioid prescribing guidelines can influence prescriber behavior, yet poor adherence continues to contribute to opioid-related issues in Swedish health care. Guideline-contrary prescribing continues to occur, as evidenced by a recent study which found that concomitant benzodiazepine dispensation was overrepresented among patients with long-term opioid treatment^16^. Lack of pain education at all levels in health care is probably also a contributing factor. There seems to exist a continued need for education initiatives and guideline dissemination in health care regarding opioid treatment in CNCP, as well as in society, and better implementation of these factors will likely improve responsible and safe opioid prescribing practices. All in all, it seems clear that the organization of the health care system, and the pain education level of its professionals, are crucial aspects which can either facilitate or hinder responsible opioid prescribing.

Lack of time has previously been pointed out as a problem when prescribing opioids^19,23^ and the prescription renewal situation was another situation which our results identified as challenging and time consuming. Similar to GPs^18^ pain specialists echoed feelings of suspicion, particularly if patients were prescribed certain opioids (e.g., fentanyl). Given the pharmacokinetic variability among opioids which may affect their analgesic and euphoric properties^57^ it is understandable that different opioids cause different reactions in pain specialists. The need for sufficient time when assessing opioid prescriptions seems to be foundational to facilitate responsible opioid prescribing, and this issue must likely be approached from a system-level perspective. Further, our results indicated that working in a team, e.g., with a nurse, was an important aspect to support the pain specialist in responsible prescribing. This, taken together with the promising results which shared decision-making and interprofessional care have shown in improving both acute and chronic pain management^58^ indicates that there are potential benefits in working in a team around the opioid-treated patient with CNCP. Sufficient time is therefore not only needed for the individual prescriber renewing the opioid prescription, but also for the interprofessional team who should be involved in the opioid-treated patient’s care.

## Strengths and limitations

To the best of our knowledge, this is the first qualitative study to specifically examine board-certified pain specialists’ experiences of prescribing opioids for CNCP. Although one participant lacked formal board certification, we think that this side-stepping from inclusion criteria has had no or minimal impact on our results. Interviewees represented all geographical areas of Swedish health care, except the Northern regions. Despite this minor lack in geographical representation, we consider our results to be transferable to Sweden and propose they could be transferable to neighboring Nordic countries, which have similar socioeconomic conditions and health care systems to Sweden. Further, our process of analysis identified results which were present in separate categories, indicating both internal validity and plausible external validity according to Malterud^59^.

## Conclusions

This study offers novel insights into what pain specialists have experienced as important relational, individual, and system level factors for safe and responsible opioid prescribing. Pain specialists and GPs appear to share many experiences, such as accounting for patient-related and relational aspects, and the challenges of treating CNCP^23^. Our participants’ experiences were similar to those found by Seangrung et al.^25^ suggesting that many experiences and perspectives may be shared by pain specialists globally.

Based on our results, we conclude that implementation of structured, biopsychosocial pain assessments in clinical practice outside of specialized pain care is necessary to improve opioid prescribing habits. Facilitating structured communication between the patient and their providers regarding the expectations, risks, and goals of opioid treatments should be prioritized. It would likely benefit patient care if health care systems and leadership addressed the perceived lack of time when prescribers assess prescription opioids in patients with CNCP in both specialized pain and primary care. Creating easy-to-access forums in which prescribers can consult each other and discuss ethical dilemmas associated with opioid prescribing would likely contribute to responsible prescribing practices and resilient physicians. Enabling interdisciplinary team-based care, continued education initiatives on the effects of long-term opioid prescribing and on available non-opioid treatment strategies, and continued development and dissemination of guidelines which supports shared-decision making within the interdisciplinary team and with the patient, are important system-level changes which may improve physician adherence to prescribing guidelines. In turn, this may help prevent problematic use of prescription opioids, potentially sparing patients from iatrogenic opioid-related suffering.

Studies examining pain specialists’ experiences of prescribing opioids for CNCP in other health care systems and geographical contexts would improve transferability of results. Exploring the perspectives of other members of the interdisciplinary team on opioid prescribing for CNCP, such as pain nurses who often are involved in the care of opioid-treated patients, would be of interest. Finally, exploring what ‘sufficient time’ means to physicians and other health care stakeholders is likely necessary to facilitate addressing this barrier to responsible opioid prescribing.

## Supplementary Information

Below is the link to the electronic supplementary material.

Supplementary Material 1

## Data Availability

The datasets generated and/or analyzed during the current study are not publicly available due to reasons of sensitivity as interviews may identify individual participants but are available from the corresponding author on reasonable request.
