# Supplementary material for: A qualitative interview study exploring the experiences of pain specialists on prescribing opioids for chronic non-cancer pain
Source: Sci Rep. 2025 Aug 13;15:29672. doi: 10.1038/s41598-025-15113-6 (PMC12350938; doi:10.1038/s41598-025-15113-6)
Supplement: Supplementary file 1 — Supplementary Material 1 [file 41598_2025_15113_MOESM1_ESM.pdf]

Supplementary Information to A qualitative interview study exploring the experiences of pain specialists on prescribing opioids for chronic non-cancer pain

Authors: Thomas F Kallman*[1], MD, Emmanuel Bäckryd[1], MD, PhD, and Anne Söderlund Schaller[1], RN, PhD

1) Pain and Rehabilitation Center, and Department of Health, Medicine and Caring Sciences, Linköping University, Linköping, Sweden.

Corresponding author:
*Thomas F Kallman
Pain and Rehabilitation Center, Brigadgatan 22, SE-581 85 Linköping, Sweden
thomas.kallman@liu.se
https://liu.se/en/employee/thoka10
+46 10 103-0000

Supplementary Methods

Please note that as all interviews were conducted in Swedish, the interview guide likewise was originally developed in Swedish. Each main question was covered in every interview. If the main questions have bullet points under them, these were probing questions which were used as needed in the interviews. Below is a translated version in English of the Swedish interview guide.

Interview guide for study

Orientation: define the situation, aim, audio and video is being recorded, are there any questions before we begin.

Fill out digital consent form

Demographic data:
Date when interview was conducted (day/month/year)
The participant's physical location at the time of the interview
Male/Female
Age
Base specialty [year for certification]
Number of years practicing as physician
Subspecialty pain medicine [year for certification]
Number of years practicing as pain specialist
Approximation of number of opioid prescriptions written per week

Interview questions

1. How do you experience prescribing opioids to patients with chronic non-cancer pain?

- Challenges? Approach? Treatment results? Doctor-patient relationship?

2. How do you view your clinic's/region's policy for opioid prescribing?

- Is there one? How is it used? Is it helpful or hindering?

3. Can you describe your experiences when you thought that a patient should not continue using opioids? Or a time when you declined prescribing/renewing a prescription?

- Thoughts (how / what / why)
- The patient?
- Help / support?
- What happens to you as a person?

4. Can any ethical problems arise when a patient should discontinue opioids?

- Can you describe an occasion when you in retrospect questioned your choice to prescribe?
- Can you tell me why you think it turned out that way in that case?
- Ethical stress?

5. Has prescribing opioids to this group of patients impacted you in your role as a physician?

- Self-image, fellow human vs medical professional

6. How do you think opioid prescribing will develop in Sweden in the coming years?

7. I have no more questions. Do you think that I should have asked different question? Have I missed anything?

8. In conclusion: Is there anything else you would like to add or ask before we end the interview?

General follow-up questions to all the above:

- *Can you develop that?*
- *Can you tell me more about x?*
- *Can you give me examples?*
- *Can you share your thought process?*
